# Effects of cigarette smoke on barrier function and tight junction proteins in the bronchial epithelium: protective role of cathelicidin LL-37

**DOI:** 10.1186/s12931-019-1226-4

**Published:** 2019-11-09

**Authors:** Miyoko Tatsuta, Keiko Kan-o, Yumiko Ishii, Norio Yamamoto, Tomohiro Ogawa, Satoru Fukuyama, Aimi Ogawa, Akitaka Fujita, Yoichi Nakanishi, Koichiro Matsumoto

**Affiliations:** 1 Research Institute for Diseases of the Chest, Graduate School of Medical Sciences, Kyushu University, 3-1-1 Maidashi, Higashi-ku, Fukuoka, 812-8582 Japan; 2 Department of Respiratory Medicine, National Hospital Organization Omuta National Hospital, Fukuoka, 837-0911 Japan; 3 Department of Endoscopic Diagnostics and Therapeutics, Kyushu University Hospital, Fukuoka, 812-8582 Japan

**Keywords:** Airway epithelial barrier function, Cigarette smoke, Glucocorticosteroid, Long-acting β_2_-agonist, Cathelicidin, LL-37

## Abstract

**Background:**

Airway epithelial barrier function is maintained by the formation of tight junctions (TJs) and adherens junctions (AJs). Inhalation of cigarette smoke causes airway epithelial barrier dysfunction and may contribute to the pathogenesis of chronic lung diseases such as asthma and chronic obstructive pulmonary disease (COPD). We assessed the effects of cigarette smoke on barrier function and expression of multiple TJ and AJ proteins in the bronchial epithelium. We also examined whether treatment with glucocorticosteroids (GCSs), long-acting β_2_-agonists (LABAs), and human cathelicidin LL-37 can protect against cigarette smoke extract (CSE)-induced barrier dysfunction.

**Methods:**

Calu-3 cells cultured at the air-liquid interface were pretreated with or without GCSs, LABAs, GCSs plus LABAs, or LL-37, and subsequently exposed to CSE. Barrier function was assessed by transepithelial electronic resistance (TEER) measurements. Gene and protein expression levels of TJ and AJ proteins were analyzed by quantitative PCR and western blotting, respectively. Immunofluorescence staining of TJ and AJ proteins was performed.

**Results:**

CSE decreased TEER and increased permeability in a concentration-dependent manner. CSE suppressed gene expression of claudin-1, claudin-3, claudin-4, claudin-7, claudin-15, occludin, E-cadherin, junctional adhesion molecule-A (JAM-A) and zonula occludens-1 (ZO-1) within 12 h post-CSE exposure, while suppressed protein expression levels of occludin at 12 h. CSE-treated cells exhibited discontinuous or attenuated immunostaining for claudin-1, claudin-3, claudin-4, occludin, ZO-1, and E-cadherin compared with untreated cells. GCS treatment partially restored CSE-induced TEER reduction, while LABA treatment had no effect. GCS and LABA combination treatment had no additive effect on CSE-induced TEER reduction and gene suppression of TJ and AJ proteins. Human cathelicidin LL-37 counteracted CSE-induced TEER reduction and prevented disruption of occludin and ZO-1. LL-37 also attenuated CSE-induced decreases in gene and protein expression levels of occludin.

**Conclusions:**

CSE caused airway epithelial barrier dysfunction and simultaneously downregulated multiple TJ and AJ proteins. GCS and LABA combination treatment had no additive effect on CSE-induced TEER reduction. LL-37 counteracted CSE-induced TEER reduction and prevented disruption of occludin and ZO-1. Use of LL-37 to counteract airway epithelial barrier dysfunction may have significant benefits for respiratory diseases such as asthma and COPD.

## Background

Asthma and chronic obstructive pulmonary disease (COPD) are chronic lung diseases associated with airway inflammation and airflow obstruction. Smoking is the most important risk factor for COPD and also has an impact on the development and severity of asthma [1, 2]. The benefits of inhaled glucocorticosteroids (GCSs) and long-acting β_2_-agonists (LABAs) in asthma and COPD are widely recognized. Clinical trials have suggested that combination treatment with inhaled GCS and LABA improves symptoms and lung function and reduces exacerbations in patients with asthma and COPD [3–5].

The bronchial epithelium act as a frontline defense against a wide range of inhaled exogenous substances. The epithelial barrier function is maintained by apical junctional complexes that form between neighboring cells and consist of apical tight junctions (TJs) underlying adherens junctions (AJs) [6]. TJ proteins, such as claudin family, occludin, junctional adhesion molecule (JAM), and zonula occludens (ZO) proteins, and AJ proteins, such as E-cadherin, have been shown to compose the junctional complexes in the bronchial epithelium [7]. In a recent study, bronchial biopsies from patients with asthma displayed patchy disruption of ZO-1 and occludin [8]. Similarly, weaker expression of ZO-1, occludin, and E-cadherin was observed in bronchial epithelium and lung tissue sections from patients with COPD compared with healthy individuals [9–11]. These findings suggest a broad defect in adhesion mechanisms in asthma and COPD. In response to cigarette smoke, acute changes in epithelial permeability and decreases in several key TJ and AJ proteins have been noted [12–14]. However, the effects of cigarette smoke exposure on multiple TJ and AJ proteins and subsequent barrier dysfunction in the bronchial epithelium are poorly understood.

Antimicrobial peptides (AMPs) comprise a large family of compounds that are considered essential for innate immunity because of their ability to kill invading respiratory pathogens as well as various other activities such as wound healing [15]. Among the AMPs, LL-37 is the only member of the cathelicidin family found in humans and was reported to upregulate the levels of TJ proteins and increase human epidermal keratinocyte barrier function [16, 17]. There is also growing evidence that LL-37 is involved in the pathogenesis of COPD [18–21]. A previous study showed that LL-37 levels in sputum were elevated in patients with COPD compared with control subjects, but decreased in asthmatic patients [22]. It remains unknown whether LL-37 is beneficial or detrimental to airway epithelial barrier function.

In this study, we evaluated the effects of cigarette smoke extract (CSE) exposure on barrier function and multiple TJ and AJ proteins in the bronchial epithelium cultured at the air-liquid interface (ALI). We also assessed the effects of GCSs (budesonide, BUD; fluticasone propionate, FP) or LABAs (salmeterol, SAL; formoterol, FOR) alone, GCS plus LABA combination treatment, and LL-37 on airway epithelial barrier function by transepithelial electrical resistance (TEER) measurements and expression of TJ and AJ proteins in the bronchial epithelium exposed to CSE.

## Methods

### Preparation of CSE

CSE was prepared as described previously [13]. Briefly, mainstream smoke from two cigarettes (Marboro brand) was bubbled through 20 ml of culture medium. After adjustment of the pH to 7.4, the CSE was sterile-filtered through a 0.22-μm filter (33-mm Millex GV; Merck Millipore, Billerica, MA). This solution was considered to be 100% CSE. CSE was standardized by measuring the absorbance at wavelength 320 nm, freshly prepared for each experiment, and diluted with culture medium supplemented with 10% FBS before use.

### Cell culture and treatment

Calu-3 cells, a sub-bronchial human epithelial cell line (HTB-55; ATCC, Manassas, VA) were cultured in Dulbecco’s modified Eagle’s medium/F-12 (Thermo Fisher Scientific, Waltham, MA) supplemented with 10% FBS and 1% penicillin-streptomycin. Cells were incubated at 37 °C in a humidified atmosphere of 95% air and 5% CO_2_.

For ALI culture, cells were seeded onto human collagen type IV-coated (Sigma, St. Louis, MO) transwell inserts (0.33-cm^2^ polyester, 0.4-μm pore size; Corning Costar, Tewksbury, MA) at a density of 1 × 10^6^ cells/cm^2^ with 200 μl apical volume and 500 μl basal volume. After 24 h, the apical medium was removed and the cells were maintained with 500 μl of culture medium in the basal chamber as described previously [23]. The basal medium was changed every other day and the monolayers were allowed to differentiate under the ALI condition for 9 days. At day 9, 200 μl of control medium or CSE was added to the apical chamber and the cells were cultured for specified times. Our preliminary experiment demonstrated that TEER in Calu-3 cells cultured under the ALI condition reached a plateau around day 8 post-seeding and then decreased to about half of maximum peak at day 21 post-seeding (Additional file 1).

In some experiments involving exposure of cells to GCS and/or LABA, cells were pretreated with 1 or 10 nM BUD (Sigma-Aldrich, St. Louis, MO) and/or 10 nM SAL (Tocris, Minneapolis, MN) or 10 nM FOR (Sigma-Aldrich), or 10 nM FP (Tocris) without or with SAL or FOR for 2 h prior to CSE exposure, by addition to the apical and basal chambers. In other experiments, 10 or 20 μg/ml LL-37 (sequence: LLGDFFRKSKEKIGKEFKRIVQRIKDFLRNLVPRTES; AnaSpec, San Jose, CA) was added to the apical and basal chambers for 2 h prior to CSE exposure. The cells were then exposed to CSE by addition to the apical chamber and incubated in CSE medium containing the pretreated reagents.

### Measurement of TEER

Bronchial epithelial cell layer integrity was evaluated by TEER measurements using a Millicell-ERS 2 V-Ohmmeter (Millipore Co., Bedford, MA) at specified time points. The medium in the apical chamber was added at 1 h before TEER measurement. The electrode was soaked in 70% ethanol and rinsed with culture medium prior to use. TEER was calculated by the following equation [24]: TEER (Ωcm^2^) = (R_sample_ – R_blank_) × effective membrane area (cm^2^).

### Permeability assay

Fluorescein isothiocyanate (FITC)-dextran (4 kDa, Sigma-Aldrich) was diluted in culture medium to a concentration of 1 mg/ml. A hundred μl of medium containing FITC-dextran and 500 μl of medium were added to the apical and basal chamber, respectively. Then cells were incubated for 2 h and then culture medium in basal chamber were collected to measure fluorescence using fluorometer (Flexstation3, Molecular Devices, Tokyo, Japan). The excitation and emission wavelengths were 488 and 525 nm, respectively. A fluorescent standard curve was generated using known concentration of FITC-dextran in culture medium.

### Trypan blue exclusion assay

After 24 h of CSE exposure, the culture medium in the apical chamber was removed, and the cells were washed with phosphate-buffered saline (PBS), detached with trypsin-EDTA, and stained with 0.4% trypan blue solution. Non-viable cells stained blue were counted in a LUNA™ Automated Cell Counter (Logos Biosystems, Annandale, VA).

### Gene expression in Calu-3 cells

Total RNA was isolated from cell lysates using TRIzol Reagent (Thermo Fisher Scientific) and cDNA was generated with a PrimeScript II First-Strand cDNA Synthesis Kit (Takara, Shiga, Japan). Real-time quantitative reverse-transcriptase PCR analyses were performed using SYBR Premix Ex Taq II (Takara) in a Thermal Cycler Dice Real Time System II (Takara) with the target gene expression levels normalized to β-actin expression. The primer sequences are provided in Additional file 2.

### Western blotting

Cells were cultured under submerged conditions in 6-well plates and grown to 100% confluence. In some experiments for analyses of phosphorylated epidermal growth factor receptor (EGFR) and extracellular signal-regulated kinase (ERK) 1/2, cells were incubated overnight in serum-free medium. Cells were lysed using Pierce^Ⓡ^ RIPA Buffer (Thermo Fisher Scientific) according to the manufacturer’s instructions. Ten micrograms of the protein sample were denatured, separated by SDS-PAGE, and transferred to polyvinylidene difluoride membranes. The membrane was incubated overnight at 4 °C with primary antibodies. The primary antibodies were as follows: rabbit anti-claudin-1 polyclonal antibody (diluted 1:250; Invitrogen, Carlsbad, CA); rabbit anti-claudin-3 polyclonal antibody (diluted 1:1000; Abcam, Cambridge, UK); mouse anti-claudin-4 monoclonal antibody (diluted 1:500; Invitrogen); rabbit anti-occludin polyclonal antibody (diluted 1:125; Thermo Fisher Scientific); rabbit anti-ZO-1 polyclonal antibody (diluted 1:250; Invitrogen); mouse anti-E-cadherin monoclonal antibody (diluted 1:500; BD Biosciences, San Jose, CA); rabbit anti-phosphorylated EGFR (Tyr1068) monoclonal antibody (diluted 1:1000; Cell Signaling Technology, Beverly, MA); rabbit anti-EGFR monoclonal antibody (diluted 1:1000; Cell Signaling Technology); rabbit anti-phosphorylated ERK 1/2 (Thr202/Tyr204) polyclonal antibody (diluted 1:1000; Cell Signaling Technology); rabbit anti-ERK 1/2 polyclonal antibody (diluted 1:1000; Cell Signaling Technology); mouse anti-β-actin monoclonal antibody (diluted 1:1000; Cell Signaling Technology). Membranes were washed and then incubated with a horseradish peroxidase-conjugated secondary antibody for 30 min at room temperature. Specific bands were visualized using ImmunoStar^Ⓡ^ LD (Wako, Osaka, Japan) according to the manufacturer’s instructions. All blots were imaged using the ChemiDoc™ XRS+ system (Bio-Rad Laboratories, Inc. Hercules, CA). Densitometric analysis of band intensities was performed using Image J.

### Immunofluorescence staining

Cells were washed with PBS, fixed with 4% paraformaldehyde for 10 min, and permeabilized with PBS containing 0.5% Triton X-100 and 3% bovine serum albumin (BSA) for 10 min. The cells were then incubated with each primary antibody prepared in PBS containing 1% BSA at 4 °C overnight, followed by incubation with Alexa Fluor 488-conjugated goat anti-rabbit IgG antibody (1:500; Abcam) or Alexa Fluor 488-conjugated goat anti-mouse IgG antibody (1:500; Invitrogen) and nuclear staining with 4′,6-diamidino-2-phenylindole (DAPI). Images of the stained cells were obtained with a confocal laser microscope (LSM700; Zeiss, Jena, Germany). The primary antibodies were as follows: rabbit anti-claudin-1 polyclonal antibody (Invitrogen); rabbit anti-claudin-3 polyclonal antibody (Abcam); mouse anti-claudin-4 monoclonal antibody (Invitrogen); rabbit anti-occludin polyclonal antibody (Thermo Fisher Scientific); rabbit anti-ZO-1 polyclonal antibody (Invitrogen); and mouse anti-E-cadherin monoclonal antibody (BD Biosciences).

### Detection of LL-37

Levels of human LL-37 (detection limit 0.1–100 ng/mL) in cell culture supernatants were determined using ELISA (Hycult Biotech, Wayne, PA). Cell supernatants were diluted 1:10 for measurement.

### Statistical analysis

Unless otherwise stated, data are expressed as means ± SEM. Comparisons of three or more sets of data were conducted by one-way ANOVA or two-way ANOVA. All statistical analyses were conducted with GraphPad Prism 8 software (GraphPad Software, San Francisco, CA). Differences were considered statistically significant at *p* < 0.05.

## Results

### CSE impairs barrier function in airway epithelial cells

**Fig. 1:**
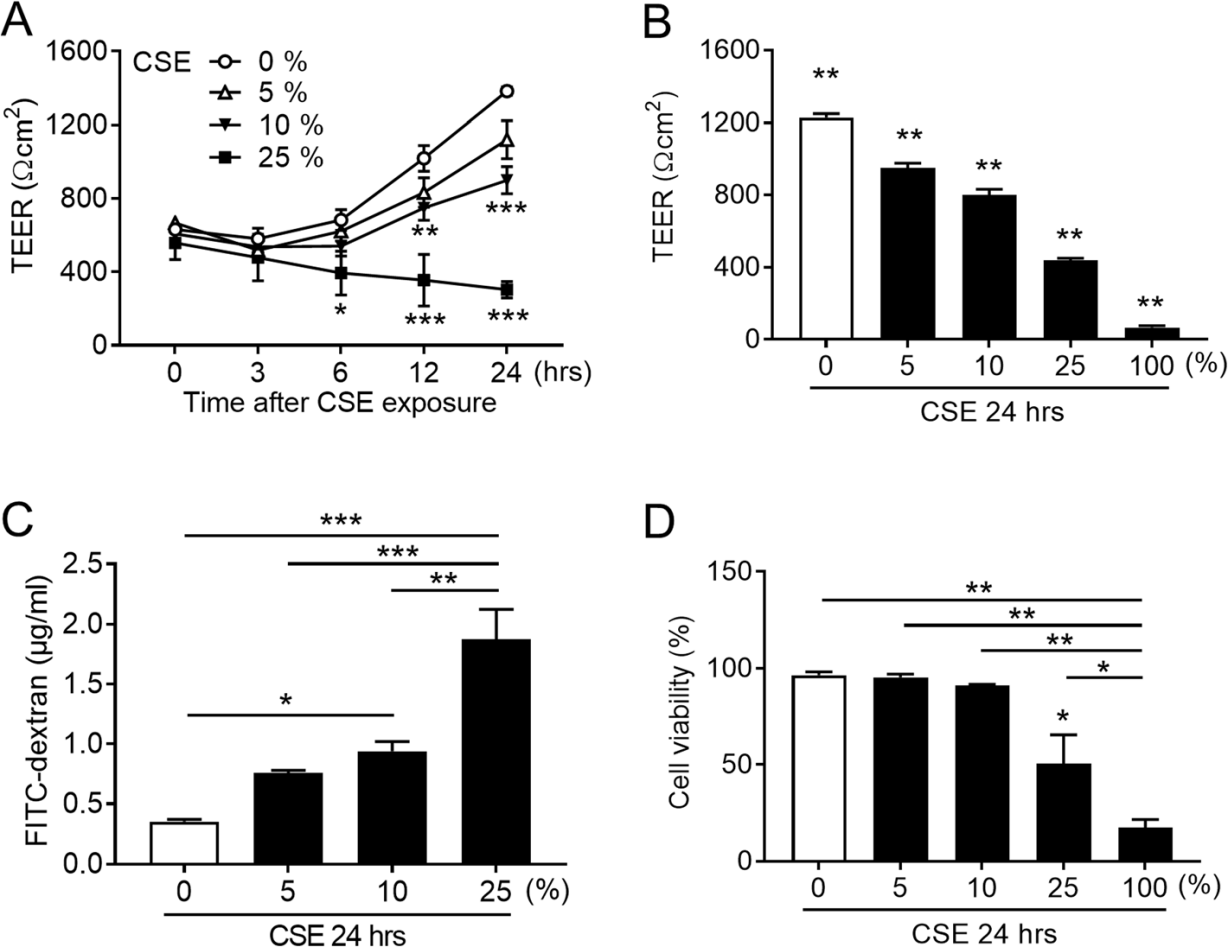
Effects of CSE on airway epithelial barrier function. **a.** Time courses of TEER in Calu-3 cells cultured in ALI and exposed to a range of CSE concentrations. **b.** Analysis of TEER from Calu-3 cells after exposure to a range of CSE concentrations for 24 h. **c.** Analysis of permeability in Calu-3 cells after exposure to a range of CSE concentrations for 24 h. **d.** Trypan blue exclusion assays for cell viability of Calu-3 cells after exposure to a range of CSE concentrations for 24 h. All results are representative of at least two independent experiments. Data represent means ± SEM (*n* = 4–9 per group). *p < 0.05, **p < 0.01, ***p < 0.001 by one- or two-way ANOVA as appropriate

To examine the effects of CSE exposure time and concentration, Calu-3 sub-bronchial epithelial cells were cultured at the ALI and epithelial barrier function was evaluated by TEER measurements and permeability assay. Cells were treated with or without a range of CSE concentrations for 0, 3, 6, 12, or 24 h, and assessed for their barrier function by TEER measurements (Fig. 1a). Treatment with CSE reduced TEER compared with untreated control cells in a concentration-dependent manner. The reduction was further exacerbated by prolonged CSE treatment. After exposure to CSE for 24 h, TEER was significantly decreased in a concentration-dependent manner and 10% CSE exposure significantly increased epithelial permeability compared with untreated control cells (Fig. 1b and c). To identify nontoxic doses of CSE for Calu-3 cells, cell viability was assessed with or without CSE exposure for 24 h by trypan blue exclusion assays, and 25% CSE was found to significantly reduce cell viability (Fig. 1d). Taking into account our requirements for sufficient reduction of TEER and an increase in permeability by CSE and minimal influence on cell viability, treatment with 10% CSE was selected for outcome measurements in subsequent experiments.

### CSE disrupts TJ and AJ proteins with suppression of gene expression levels in airway epithelial cells

**Fig. 2:**
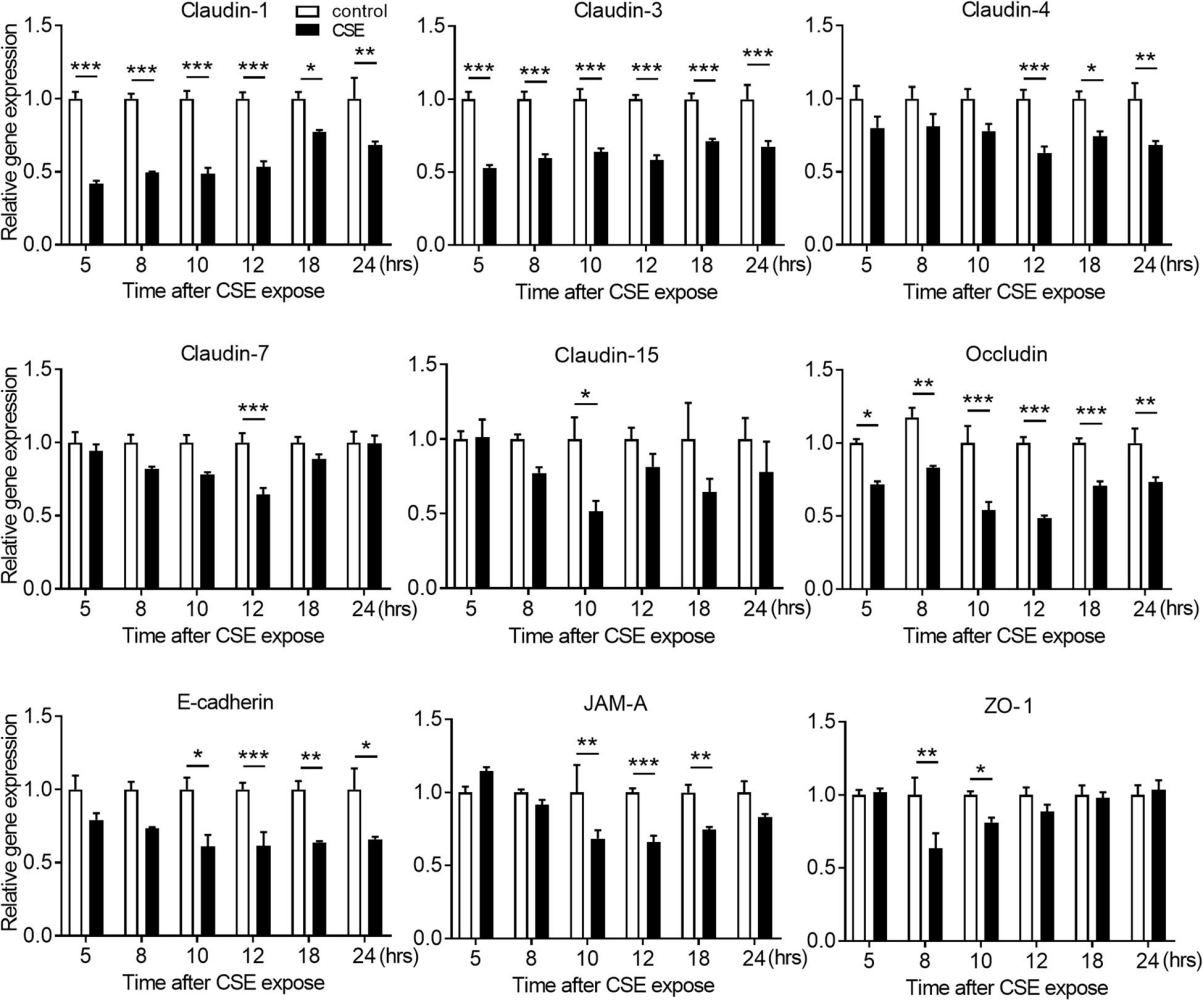
Effects of CSE on the gene expression levels of TJ and AJ proteins in Calu-3 cells. Real-time quantitative reverse-transcriptase PCR analyses of gene expression levels for TJ and AJ proteins in Calu-3 cells exposed or unexposed to 10% CSE for the indicated times. Data were normalized to β-actin expression and expressed as means ± SEM (*n* = 4–6 per group). All results are representative of at least two independent experiments. *p < 0.05, **p < 0.01, ***p < 0.001, by two-way ANOVA

**Fig. 3:**
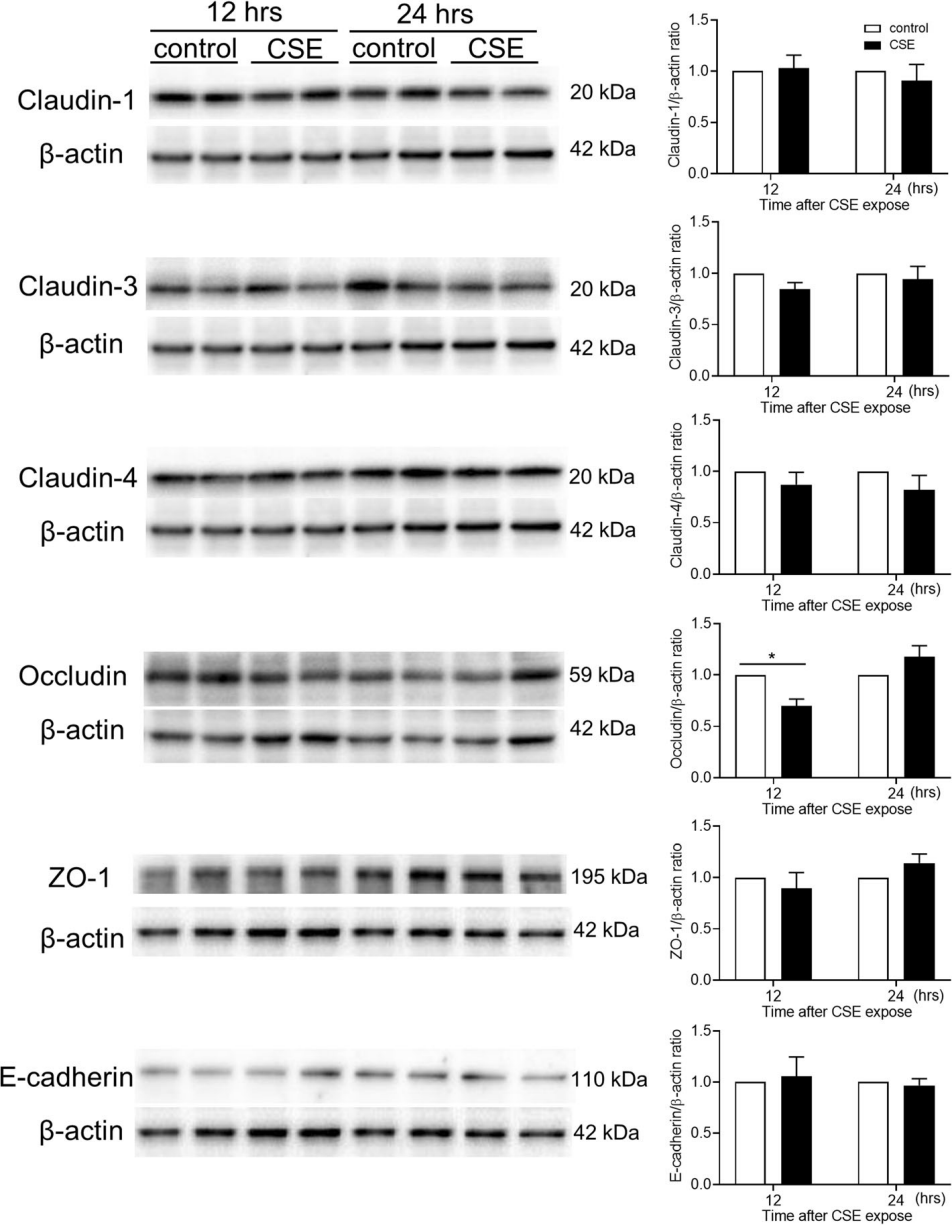
Representative western blots showing TJ and AJ proteins in Calu-3 cells exposed or unexposed to 10% CSE for 12 or 24 h. Band intensity was quantitated using densitometry. All results are representative of at least two independent experiments. Data represent means ± SEM (*n* = 6–12 per group). *p < 0.01 by two-way ANOVA

**Fig. 4:**
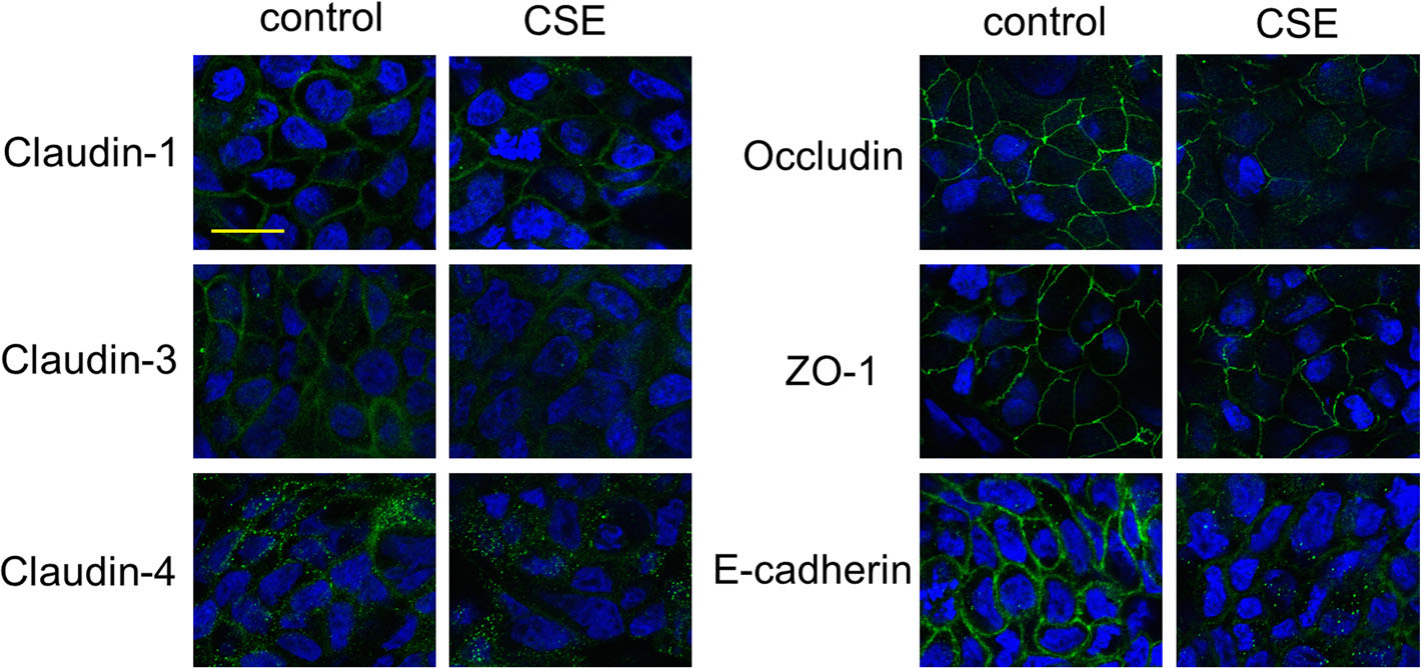
Representative confocal immunofluorescence images of TJ and AJ proteins in Calu-3 cells exposed or unexposed to 10% CSE for 24 h. Staining of claudin-1, claudin-3, claudin-4, occludin, ZO-1, and E-cadherin is shown in green, and DAPI staining is shown in blue. Scale bar, 10 μm. All results are representative of at least two independent experiments

We investigated the effects of CSE exposure on TJ proteins (claudins, occludin, JAM-A, and ZO-1) and an AJ protein (E-cadherin). Calu-3 cells were exposed or unexposed to 10% CSE and the gene and protein expression levels of TJ and AJ proteins were analyzed by quantitative PCR and western blotting of total cell lysates, respectively. CSE significantly decreased the gene expression levels of claudin-1, claudin-3 and occludin at 5 h after CSE exposure compared with untreated control cells, and significantly decreased ZO-1 gene expression at 8 h and claudin-15, E-cadherin and JAM-A gene expression at 10 h (Fig. 2). Gene expression levels of claudin-4 and claudin-7 were decreased at 12 h after exposure, while suppression of claudin-15 and ZO-1 gene expression was lost within 12 h (Fig. 2). Western blotting showed that protein levels in occludin were decreased at 12 h after exposure compared with untreated control cells, but not in claudin-1, claudin-3, claudin-4, ZO-1 and E-cadherin (Fig. 3). Immunofluorescence staining of Calu-3 cells exposed or unexposed to CSE were analyzed using confocal microscopy. Immunofluorescence for claudin-1, claudin-3, claudin-4, occludin, E-cadherin, and ZO-1 revealed continuous staining in untreated control cells (Fig. 4). Meanwhile, CSE-treated cells demonstrated discontinuous or attenuated staining of these proteins at 24 h after CSE exposure (Fig. 4), indicating disruption of TJs and AJs.

### Effects of LABA treatment alone or combination treatment with LABA and GCS on CSE-induced TEER reduction in airway epithelial cells

GCSs were reported to exert protective effects on CSE-induced barrier dysfunction in vitro [25]. However, the effect of GCS and LABA combination treatment on airway epithelial barrier function remains unclear. We investigated the effects of LABA treatment alone or combination treatment with LABA and GCS on CSE-induced reduction in TEER.

**Fig. 5:**
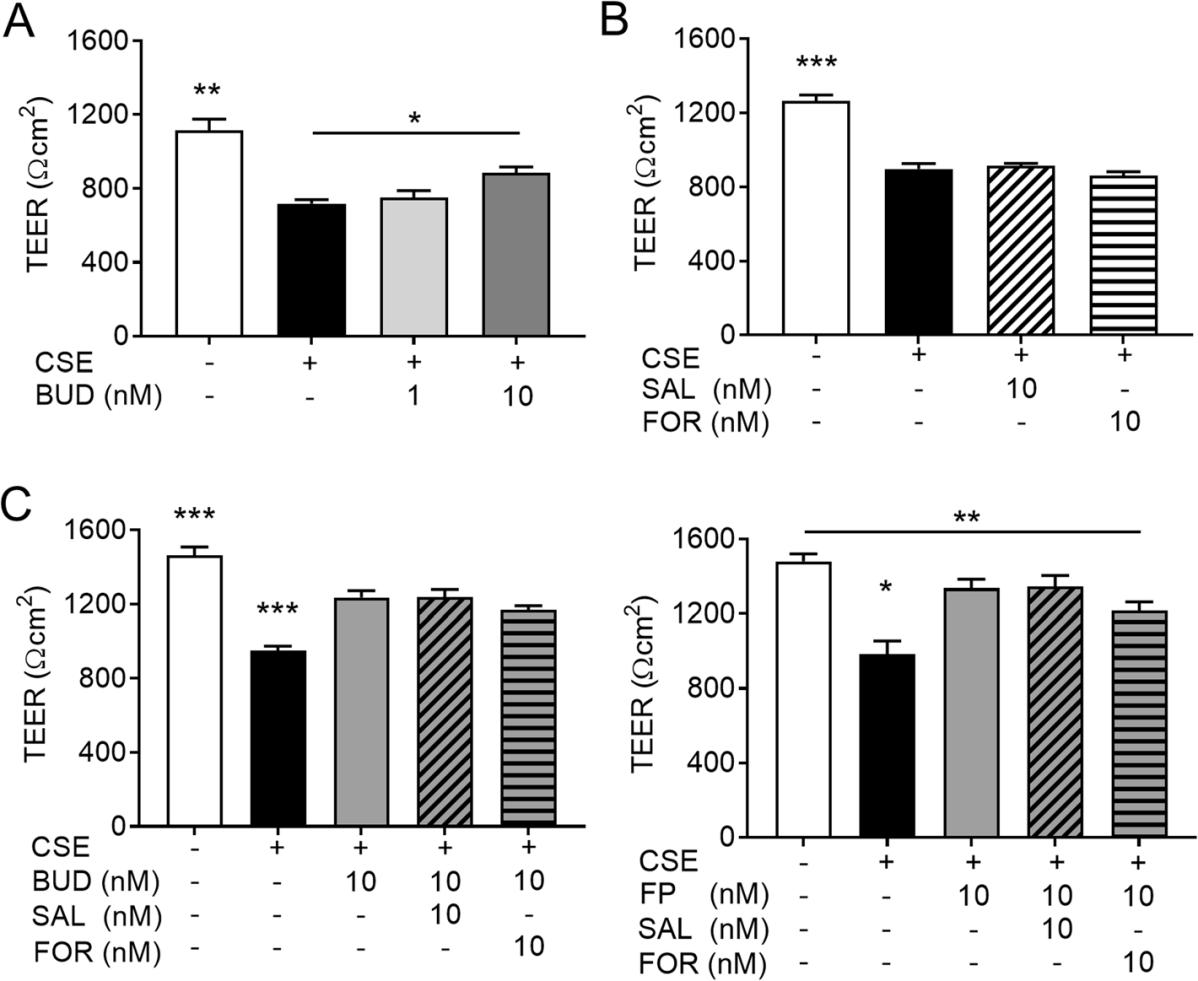
Effects of GCSs and LABAs on CSE-induced TEER reduction in Calu-3 cells. **a**. Dose-dependent protective effects of BUD on TEER on Calu-3 cells exposed to 10% CSE for 24 h. **b.** No effects of SAL or FOR on Calu-3 cells exposed to 10% CSE for 24 h. **c.** No additive effects of LABAs in combination with GCSs on TEER in Calu-3 cells exposed to 10% CSE for 24 h. All results are representative of at least two independent experiments. Data represent means ± SEM (*n* = 8–16 per group). *p < 0.05, **p < 0.01, ***p < 0.001 by one-way ANOVA

Calu-3 cells were treated with or without 1 or 10 nM BUD for 2 h and subsequently exposed to 10% CSE in medium containing BUD for 24 h. This concentration of BUD was previously shown to reflect the therapeutic levels achieved during inhalation in the human lung [26]. The CSE-induced reduction in TEER (mean, 718.8 Ωcm^2^) was partially, although significantly, attenuated in cells treated with 10 nM BUD (mean, 887.8 Ωcm^2^) (Fig. 5a). However, treatment with 10 nM SAL or FOR alone did not change the TEER in Calu-3 cells exposed to CSE (Fig. 5b). Treatment with LABA in combination with 10 nM BUD had no additive effect on CSE-induced reduction in TEER (Fig. 5c, left panel). Furthermore, 10 nM FP, another inhaled GCS, partially recovered the CSE-induced reduction in TEER, while treatment with LABA in combination with FP showed no additive effect (Fig. 5c, right panel), similar to treatment with LABA plus BUD. We confirmed that treatment with higher concentrations of BUD, SAL, or FOR without CSE exposure did not change the TEER compared with untreated cells (Additional file 3).

**Fig. 6:**
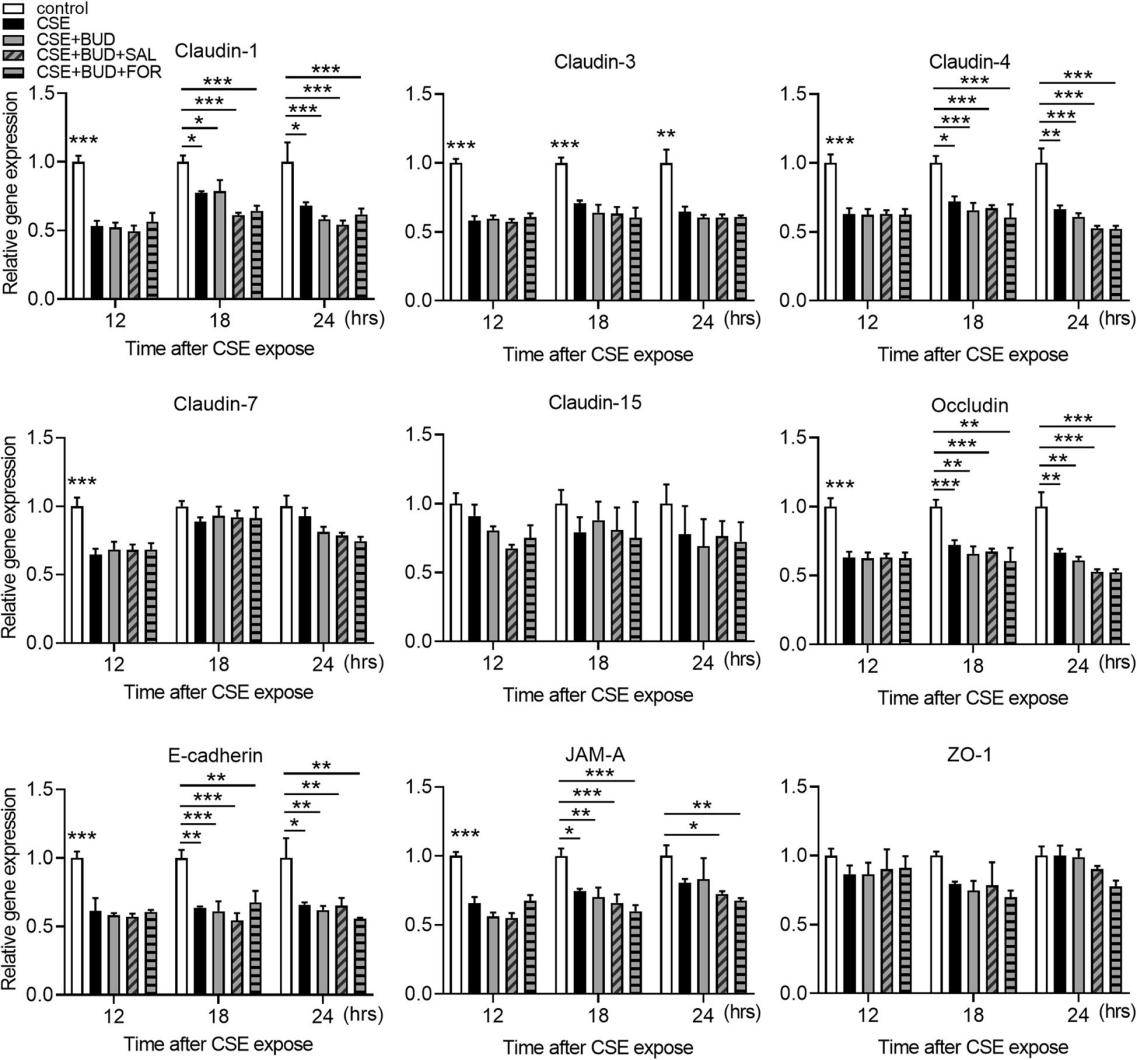
No additive effects of LABAs in combination with BUD on gene expression levels of TJ and AJ proteins in Calu-3 cells. Cells were exposed to 10% CSE for the indicated times and then real-time quantitative reverse-transcriptase PCR was performed. The target gene expression levels were normalized by β-actin expression and presented as means ± SEM (*n* = 4–8 per group). All results are representative of at least two independent experiments. *p < 0.05, **p < 0.01, ***p < 0.001 by two-way ANOVA

We further examined the effect of BUD alone or in combination with LABA on the gene expression levels for TJ and AJ proteins in cells exposed to CSE for 12, 18 and 24 h. Again, CSE decreased the levels of claudin-1, claudin-3, claudin-4, claudin-7, occludin, E-cadherin, and JAM-A gene expression compared with control cells, but did not affect claudin-15 and ZO-1 gene expression at the indicated time points (Fig. 6). Although BUD attenuated CSE-induced reduction in TEER, treatment with BUD alone or in combination with SAL or FOR did not attenuate the gene expression levels for TJ and AJ proteins suppressed by CSE (Fig. 6).

### Effects of LL-37 on CSE-induced TEER reduction in airway epithelial cells

**Fig. 7:**
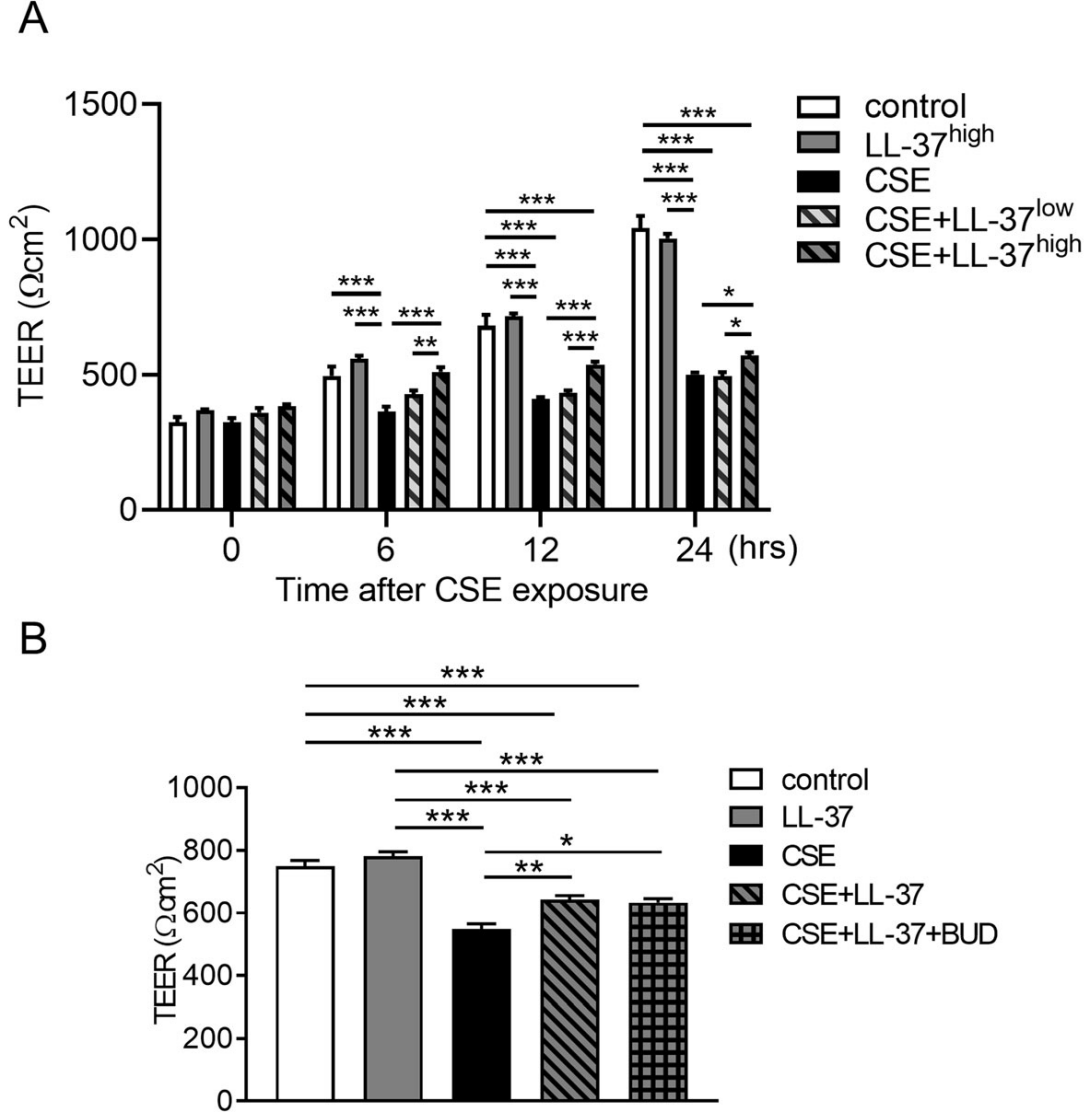
Effects of LL-37 on CSE-induced TEER reduction in Calu-3 cells. **a**. Dose-dependent protective effects of LL-37 on CSE-induced TEER reduction. Cells were pretreated with or without LL-37 and then exposed to 10% CSE. TEER was measured at the indicated times. LL-37^high^: 20 μg/ml; LL-37^low^: 10 μg/ml. **b.** No additive effects of LL-37 in combination with BUD on TEER in Calu-3 cells exposed to 10% CSE. Cells were pretreated with 20 μg/ml LL-37 in combination with or without 10 nM BUD and then exposed to CSE for 12 h. All results are representative of at least two independent experiments. Data represent means ± SEM (*n* = 6–9 per group). *p < 0.05, **p < 0.01, ***p < 0.001 by one- or two-way ANOVA as appropriate

**Fig. 8:**
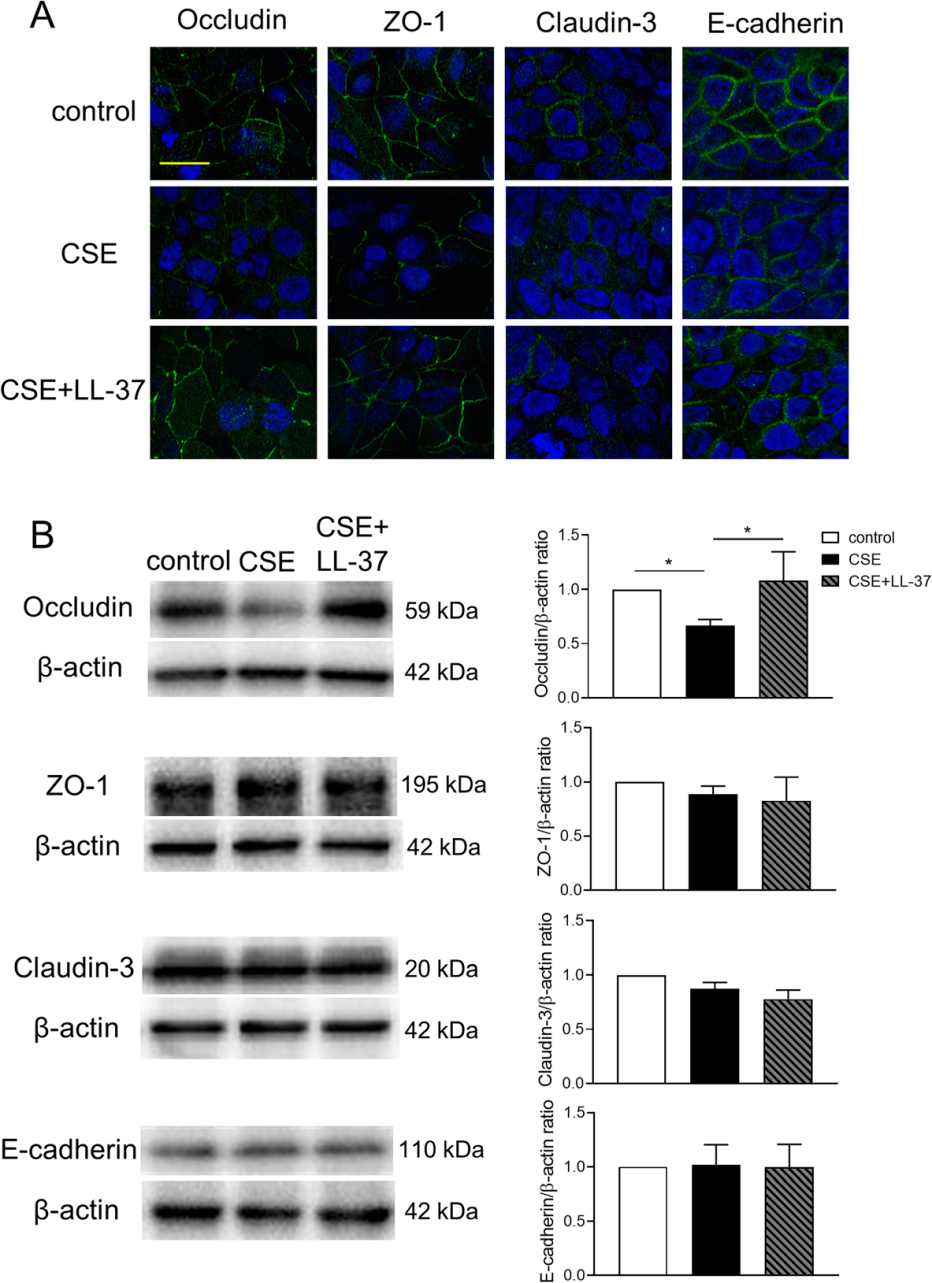
Effects of LL-37 on protein expression of TJ and AJ proteins in Calu-3 cells. Cells pretreated with or without 20 μg/ml LL-37 were exposed to 10% CSE for 12 h. **a.** Representative confocal immunofluorescence images of the effects of LL-37 on CSE-induced disruption of TJ and AJ proteins. Staining of occludin, ZO-1, claudin-3, and E-cadherin is shown in green, and DAPI staining is shown in blue. Scale bar, 10 μm. **b.** Representative western blots showing TJ and AJ proteins in Calu-3 cells. Band intensity was quantitated using densitometry. All results are representative of at least two independent experiments. Data represent means ± SEM (*n* = 4–6 per group). *p < 0.05 by one-way ANOVA

**Fig. 9:**
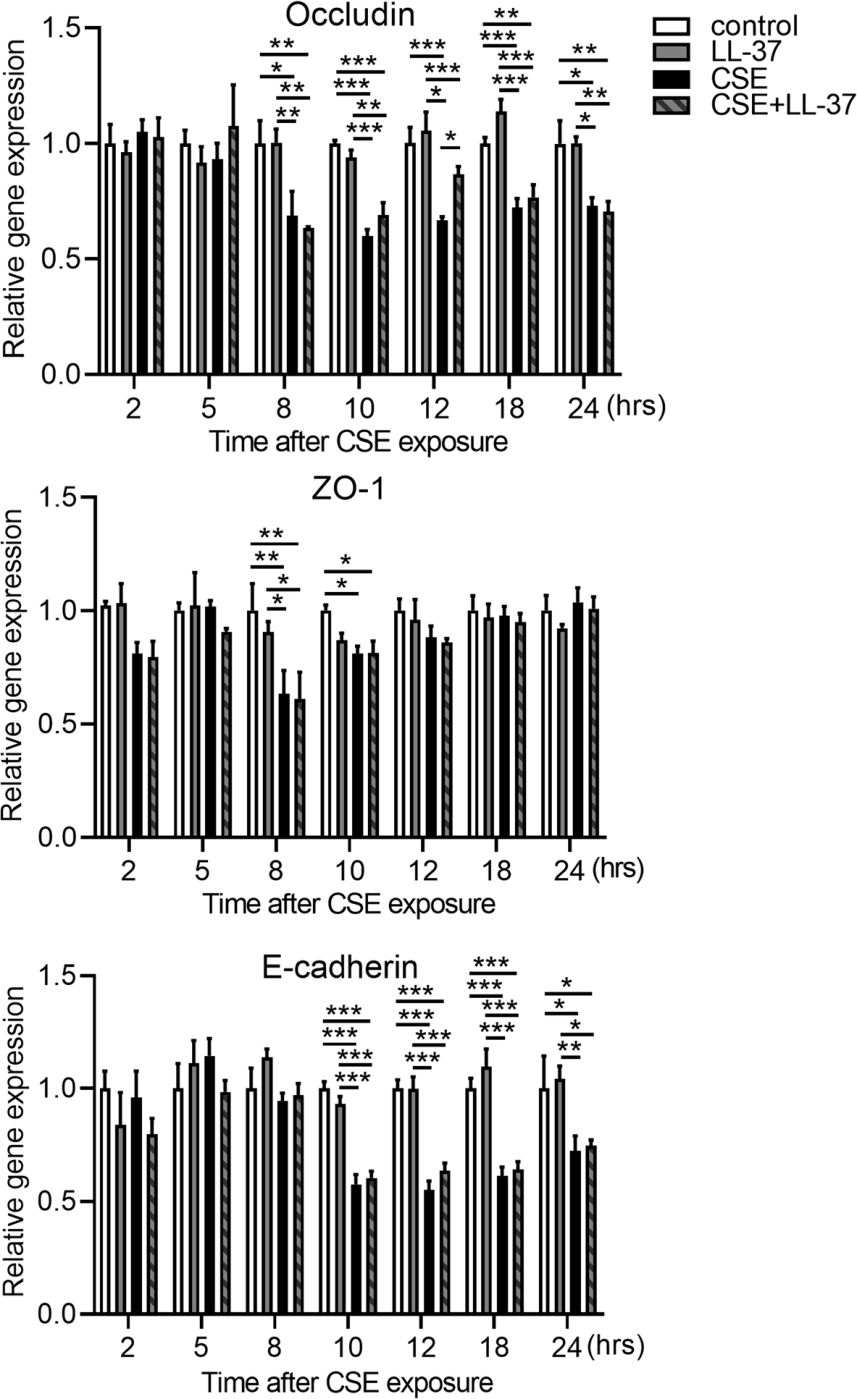
Effects of LL-37 on gene expression levels for occludin, ZO-1, and E-cadherin in Calu-3 cells. Cells were pretreated with or without 20 μg/ml LL-37 and then exposed to 10% CSE. RNA was extracted at the indicated times after CSE exposure and real-time quantitative reverse-transcriptase PCR analyses were performed. The target gene expression levels were normalized by β-actin expression and presented as means ± SEM (*n* = 4–8 per group). All results are representative of at least two independent experiments. *p < 0.05, **p < 0.01, ***p < 0.001, by two-way ANOVA

Finally, we investigated whether human cathelicidin LL-37 can diminish CSE-induced reduction in TEER and disruption of TJs and AJs in the bronchial epithelium. We firstly confirmed that production of very low levels of LL-37 (approximately 15–30 ng/ml) was induced by CSE exposure in apical and basal culture medium and returned to the control levels within 18 h after CSE exposure (Additional file 4). Calu-3 cells were pretreated with or without 10 (low) or 20 (high) μg/ml LL-37 for 2 h and then exposed to 10% CSE medium containing LL-37. TEER measurements were performed for up to 24 h after CSE stimulation. Treatment with the high concentration of LL-37 in CSE-unexposed cells did not change the TEER compared with untreated control cells. The CSE-induced reduction in TEER was significantly attenuated in cells treated with 20 μg/ml LL-37 at 6, 12, and 24 h after CSE exposure (Fig. 7a). This beneficial effect of LL-37 on CSE-induced reduction in TEER was lost at 48 h after CSE exposure with or without retreatment with 20 μg/ml LL-37 at 24 h after exposure (Additional file 5). Treatment with LL-37 in combination with 10 nM BUD had no additive protective effect on CSE-induced reduction in TEER (Fig. 7b). Next, we assessed whether LL-37 can protect against CSE-induced disruption of representative TJ and AJ proteins in the bronchial epithelium using immunofluorescence microscopy. After 12 h of CSE exposure, disrupted junctional expression of occludin and ZO-1 was observed and membrane staining of claudin-3 and E-cadherin was modestly disrupted (Fig. 8a). Treatment with LL-37 prevented the CSE-induced disruption of occludin and ZO-1, but not claudin-3 and E-cadherin (Fig. 8a). Western blotting showed increased protein level of occludin in cells treated with LL-37 compared with CSE-exposed cells without LL-37 treatment at 12 h after CSE exposure, but not at 24 h (Fig. 8b and Additional file 6). LL-37 treatment did not affect the protein levels of ZO-1, claudin-3 and E-cadherin at 12 h and 24 h after exposure (Fig. 8b and additional file 6). The effects of LL-37 treatment on the gene expression levels for occludin, ZO-1, and E-cadherin were analyzed. LL-37 treatment in CSE-exposed cells increased gene expression levels of occludin compared with cells exposed to CSE without LL-37 treatment at 12 h after CSE exposure, but not ZO-1 and E-cadherin (Fig. 9). Treatment with LL-37 alone did not change the gene expression levels of occludin, ZO-1 and E-cadherin compared with untreated control cells (Fig. 9).

## Discussion

The bronchial epithelium is responsible for preserving airway homeostasis in the lung. Disruption of the barrier integrity enhances translocation of inhaled particles into the subepithelial space where they encounter innate immune cells and cause airway inflammation and immune responses, and thus dysfunction of epithelial junctions is increasingly linked to airway diseases [7, 10]. Our experiments on the bronchial epithelium revealed that CSE exposure caused disruption of TJ and AJ proteins, with subsequent airway epithelial barrier dysfunction. Clinically relevant GCS concentrations partially restored the CSE-induced TEER reduction in airway epithelial cells, while LABAs had no effect. GCS and LABA combination treatment had no additive effect on CSE-induced reduction in TEER and suppressed gene expression levels for TJ and AJ proteins. Finally, human cathelicidin LL-37 counteracted the CSE-induced TEER reduction and disruption of occludin and ZO-1. LL-37 treatment also attenuated CSE-induced decreases in gene and protein levels of occludin.

Previous studies showed that CSE induced disassembly of ZO-1, ZO-2, occludin, or E-cadherin in the bronchial epithelium [12–14]. To the best of our knowledge, this is the first demonstration that cigarette smoke simultaneously decreased multiple TJ and AJ proteins at gene expression and fluorescence intensity levels and caused airway epithelial barrier dysfunction. TJ proteins and E-cadherin gene expression levels were suppressed within 12 h after CSE exposure, followed by discontinuous or attenuated immunofluorescence staining of TJ proteins and E-cadherin without decreases in total protein levels of TJ proteins and E-cadherin except for occludin. Previous studies also showed that alteration of TJ proteins distribution in immunofluorescence microscopy was not accompanied by changes in TJ proteins levels analyzed using western blotting, suggesting that changes in permeability may be due to alteration in the organization of TJ and AJ proteins rather than changes in their protein expression levels [24, 25, 27]. Petecchia et al [28] reported that damage to the bronchial epithelium following cigarette smoke exposure induced disassembly of ZO-1, modulated through EGFR-ERK 1/2 signaling pathway. Our study demonstrated that CSE exposure in Calu-3 cells induced phosphorylation of ERK 1/2 within 5 min after exposure, although phosphorylation of EGFR was observed in untreated control cells and further phosphorylation of EGFR was not induced by CSE exposure (Additional file 7A). Furthermore, cigarette smoke exposure and subsequent reactive oxygen species (ROS) production were shown to induce EGFR phosphorylation in the bronchial epithelium [29]. These findings suggest that ROS produced after cigarette smoke exposure may induce disassembly of multiple TJ and AJ proteins and impair airway epithelial barrier function, resulting in sustained inflammatory responses that propagate into the subepithelial tissue and lead to disease progression in asthma and COPD. Mitchell et al [30] reported differential effects of claudin-3 and claudin-4 on alveolar epithelial barrier function in rats. Further studies are needed to clarify the detailed functional roles of individual TJ and AJ proteins in regulating airway epithelial barrier function.

Many studies have demonstrated the clinical benefits of adding LABA to inhaled GCS, and inhaled GCS/LABA combination therapies are currently used worldwide for the treatment of asthma and COPD [3, 5, 31]. Molecular interactions between GCSs and β_2_-adrenoceptors have been reported [32]. β_2_-agonists induce glucocorticoid receptor (GR) nuclear translocation and alter GR phosphorylation, while GCSs increase the transcription of β_2_-adrenoceptors [33–35]. However, in our observations, GCS and LABA combination treatment had no additive effect on CSE-induced reduction in TEER. FOR in combination with BUD or FP, although not significant, counteracted the protective effect of GCS treatment on CSE-induced reduction in TEER. This protective effect of GCS treatment is similar to the findings by Heijink et al [25], who described that 16 nM BUD almost completely prevented the 7.5% CSE-induced defect in barrier function in 16HBE cells. They also reported that GCS treatment attenuated the disruption of ZO-1 following CSE exposure evaluated by immunofluorescence staining, but did not significantly affect the total protein expression of ZO-1. We also demonstrated that GCS with or without LABA did not affect the gene expression levels of TJ and AJ proteins suppressed by CSE exposure. These data suggest that transcriptional regulation of TJ and AJ proteins may not contribute to the observed effects of GCSs on airway epithelial barrier function, and this may be the reason why GCS and LABA combination treatment did not have an additive effect.

Human cathelicidin LL-37 produced by neutrophils, macrophages, and various epithelial cells plays an important role in host defense against infection and inflammation [36]. Akiyama et al [17] reported that the same concentration of LL-37 as a single treatment increased human epidermal keratinocyte barrier function through upregulation of gene and protein expression levels for multiple TJ proteins. However, our results showed that LL-37 treatment without CSE did not affect either the barrier function by TEER measurements or gene expression levels of TJ and AJ proteins in the bronchial epithelium. These findings suggest that there may be cell type-specific gene regulation of TJ proteins by LL-37. There is growing evidence of an association between LL-37 and the pathogenesis of COPD [18–21]. High sputum LL-37 levels in stable COPD were associated with increased risk of exacerbations, non-typeable *Haemophilus influenzae* colonization, and high levels of inflammatory markers [20]. Meanwhile, plasma LL-37 levels in patients with COPD were lower than those in control subjects, and LL-37 level and FEV_1_ were positively correlated in the severe COPD group [21]. The LL-37 levels in bronchoalveolar lavage fluid were elevated in patients with COPD at GOLD I–II stage, but reduced in patients with advanced COPD at GOLD III–IV stage [19]. Despite this evidence for an association between LL-37 and COPD, there have been no reports on the effect of LL-37 on airway epithelial barrier function. We demonstrated that 1000 times higher concentration of LL-37 than CSE-induced endogenous LL-37 completely attenuated CSE-induced reduction in TEER at 6 h post-CSE exposure and that this protective effect was sustained for up to 24 h. Furthermore, the attenuation of CSE-induced reduction in TEER by LL-37 was accompanied by decreases in occludin and ZO-1 disruption evaluated by immunofluorescence staining. LL-37 also attenuated CSE-induced decreases in gene and protein expression levels of occludin. In addition to its antibacterial activity, LL-37 may protect the airway epithelial barrier function against cigarette smoke and is a potential therapeutic agent for lung diseases. The mechanism for the protective effects of LL-37 remains unknown. It is reported that LL-37 transactivates the EGFR in human airway epithelial cells [37]. However, in our observation, LL-37 treatment did not affect the phosphorylation of EGFR and ERK in CSE-exposed cells (Additional file 7B).

A limitation of our study is that experiments were performed using only a single cell line Calu-3. Usefulness of Calu-3 as a model of airway epithelium has been well known due to its formation of polarized epithelial cell layer and acquisition of features such as mucus production, transport and metabolic systems, while typical ciliated cell phenotypes seen in primary bronchial epithelial cell culture are not produced in Calu-3 even under the ALI condition [38–40]. Further investigation is needed to evaluate the effect of LL-37 on airway barrier function using primary bronchial epithelial cells collected from multiple donors.

## Conclusions

Taken together, our results showed that CSE exposure to the bronchial epithelium led to airway epithelial barrier dysfunction and simultaneous downregulation of multiple TJ and AJ proteins. Clinically relevant GCS concentrations partially attenuated the CSE-induced reduction in TEER without significant changes in gene expression levels for TJ and AJ proteins, although GCS and LABA combination treatment had no additive effect on CSE-induced reduction in TEER. Finally, human cathelicidin LL-37 counteracted the CSE-induced reduction in TEER and prevented disruption of occludin and ZO-1. LL-37 also attenuated CSE-induced decreases in gene and protein expression levels of occludin. Use of LL-37 to counteract airway epithelial barrier dysfunction may have significant benefits for lung diseases such as asthma and COPD.

## Supplementary information

**Additional file 1:** Development of TEER in Calu-3 cells grown at ALI. Cells were cultured under the ALI condition over 35 days and TEER was measured every 1–3 days. Data represent means ± SEM of three replicates from an experiment.

**Additional file 2:** Sequences of real-time PCR primers used in this study.

**Additional file 3: **Effects of BUD or LABAs alone on TEER in Calu-3 cells. Cells were treated with BUD, SAL, or FOR at the indicated concentrations for 24 h and TEER was measured. All results are representative of at least two independent experiments. Data represent means ± SEM (*n* = 3–7 per group). Differences in data were analyzed by one-way ANOVA.

**Additional file 4: **CSE-induced production of LL-37 in culture medium. Calu-3 cells were exposed to 10% CSE and then cell culture supernatants in apical and basal chambers were collected at 8, 18, 24 h after CSE exposure. The concentration of LL-37 in culture supernatants were measured by ELISA. All results are representative of at least two independent experiments. Data represent means ± SEM (*n* = 5 per group). *p < 0.01, **p < 0.001, by two-way ANOVA.

**Additional file 5:** No protective effects of LL-37 on CSE-induced reduction in TEER disappeared at 48 h after CSE exposure. Calu-3 cells pretreated with or without 20 μg/ml LL-37 were exposed to 10% CSE. **A.** TEER was measured at 48 h after CSE exposure without retreatment with LL-37. **B.** Retreatment with 20 μg/ml LL-37 without replacement of the medium was performed at 24 h after CSE exposure and then TEER was measured at 48 h. All results are representative of at least two independent experiments. Data reresent means ± SEM (*n* = 6–8 per group). *p < 0.001 by one-way ANOVA.

**Additional file 6: **The effects of LL-37 on protein expression levels for TJ and AJ proteins in Calu-3 cells. Representative western blots showing TJ and AJ proteins in cells pretreated with or without 20 μg/ml LL-37 were exposed to 10% CSE for 24 h. Band intensity was quantitated using densitometry. All results are representative of at least two independent experiments. Data are means ± SEM (*n* = 3–4 per group). Differences in data were analyzed by one-way ANOVA.

**Additional file 7: **CSE-induced phosphorylation of ERK and effects of LL-37 on phosphorylation of EGFR and ERK. **A.** Representative western blots showing phosphorylated EGFR, EGFR, phosphorylated ERK or ERK in Calu-3 cells exposed to 10% CSE for 0, 5 or 10 min after CSE exposure. **B.** Representative western blots showing phosphorylated EGFR, EGFR, phosphorylated ERK or ERK in Calu-3 cells pretreated with or without 20 μg/ml LL-37 and then exposed to 10% CSE for 5 or 10 min after CSE exposure. Band intensity was quantitated using densitometry. All results are representative of at least two independent experiments. Data represent means ± SEM (*n* = 4 per group). Differences in data were analyzed by two-way ANOVA.

## Data Availability

Not applicable.

## Abbreviations

AJ: Adherens junction
ALI: Air-liquid interface
AMP: Antimicrobial peptide
BUD: Budesonide
COPD: Chronic obstructive pulmonary disease
CSE: Cigarette smoke extract
EGFR: Epidermal growth factor receptor
ERK: Extracellular signal-regulated kinase
FOR: Formoterol
FP: Fluticasone propionate
GCS: Glucocorticosteroid
GR: Glucocorticoid receptor
JAM: Junctional adhesion molecule
LABA: Long-acting β_2_-agonist
ROS: Reactive oxygen species
SAL: Salmeterol
TEER: Transepithelial electrical resistance
TJ: Tight junction
ZO: Zonula occludens
