# Supplementary figures and images for: Effects of cigarette smoke on barrier function and tight junction proteins in the bronchial epithelium: protective role of cathelicidin LL-37
Source: Respir Res. 2019 Nov 9;20:251. doi: 10.1186/s12931-019-1226-4 (PMC6842552; doi:10.1186/s12931-019-1226-4)

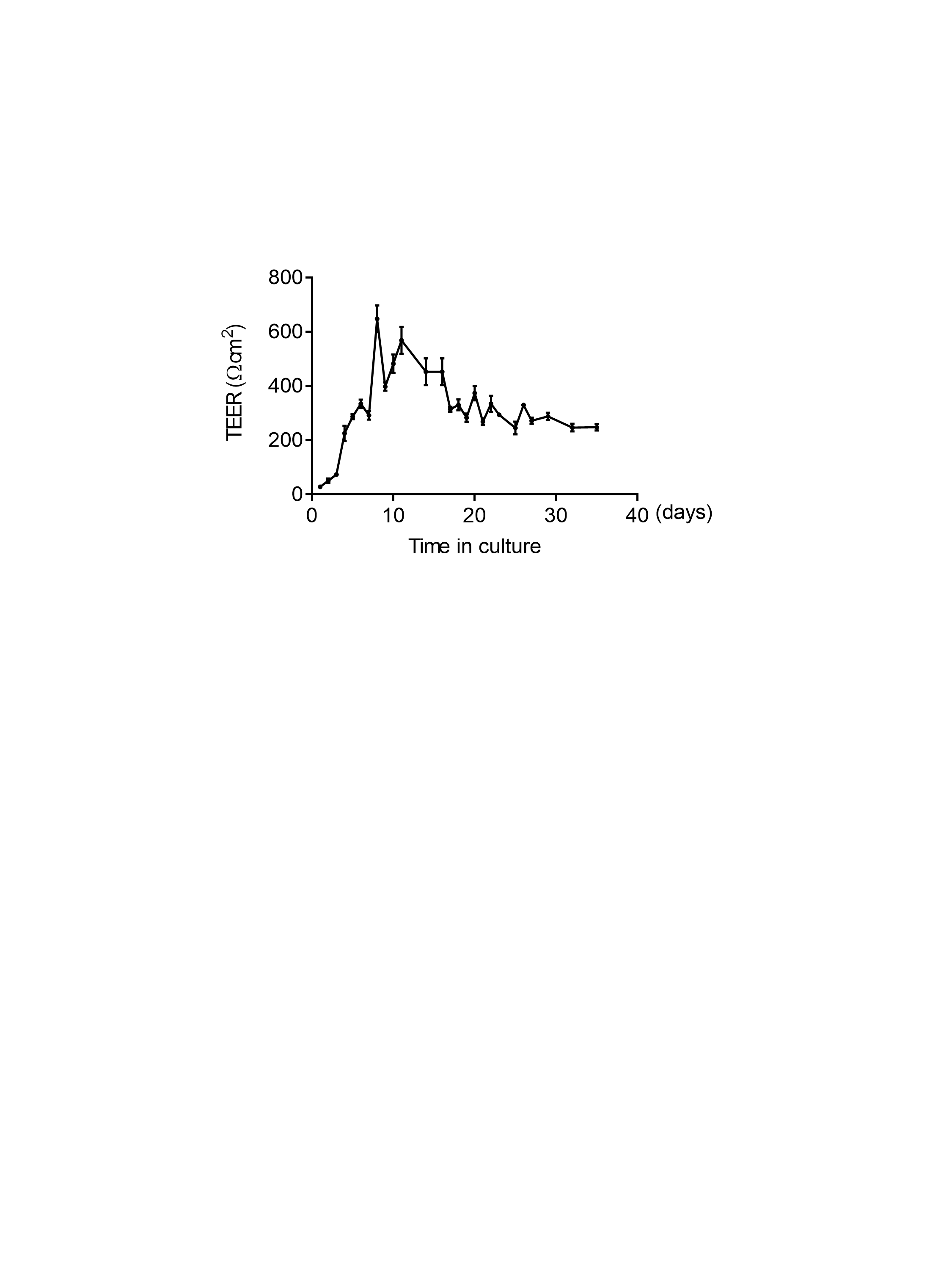

Supplement: Supplementary file 1 — Additional file 1: Development of TEER in Calu-3 cells grown at ALI. Cells were cultured under the ALI condition over 35 days and TEER was measured every 1–3 days. Data represent means ± SEM of three replicates from an experiment. [file 12931_2019_1226_MOESM1_ESM.tif]

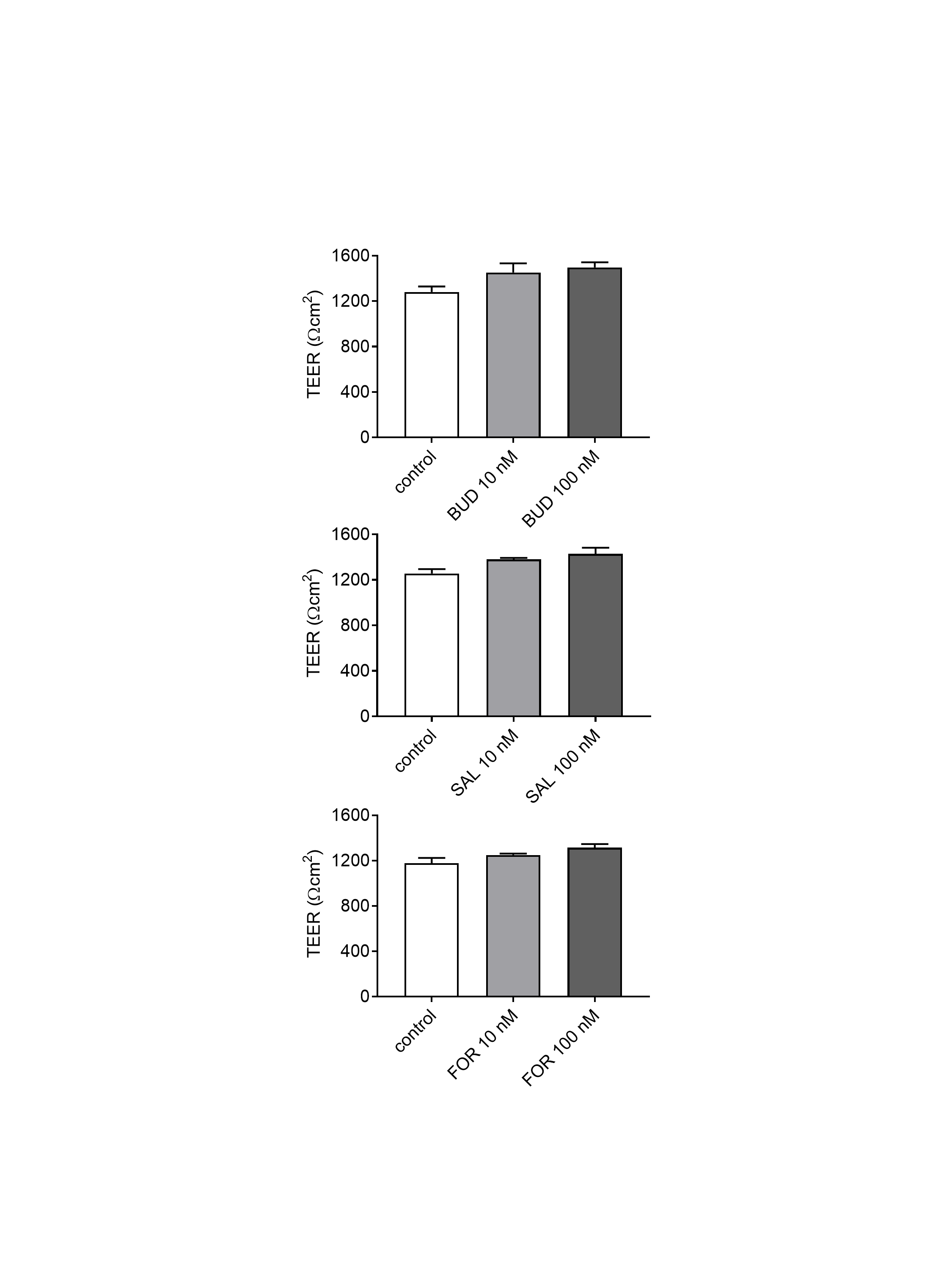

Supplement: Supplementary file 3 — Additional file 3: Effects of BUD or LABAs alone on TEER in Calu-3 cells. Cells were treated with BUD, SAL, or FOR at the indicated concentrations for 24 h and TEER was measured. All results are representative of at least two independent experiments. Data represent means ± SEM (n = 3–7 per group). Differences in data were analyzed by one-way ANOVA. [file 12931_2019_1226_MOESM3_ESM.tif]

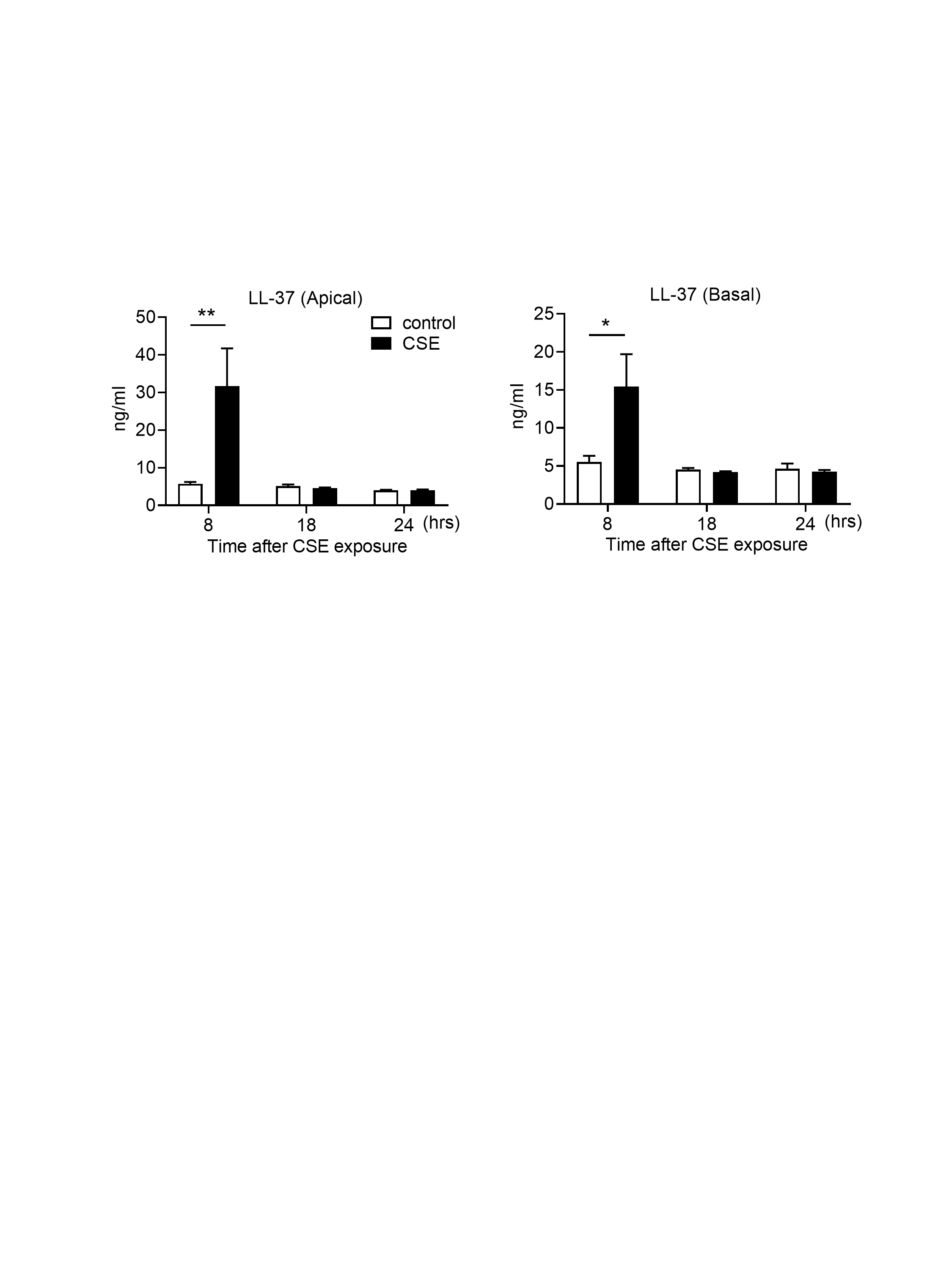

Supplement: Supplementary file 4 — Additional file 4: CSE-induced production of LL-37 in culture medium. Calu-3 cells were exposed to 10% CSE and then cell culture supernatants in apical and basal chambers were collected at 8, 18, 24 h after CSE exposure. The concentration of LL-37 in culture supernatants were measured by ELISA. All results are representative of at least two independent experiments. Data represent means ± SEM (n = 5 per group). *p < 0.01, **p < 0.001, by two-way ANOVA. [file 12931_2019_1226_MOESM4_ESM.tif]

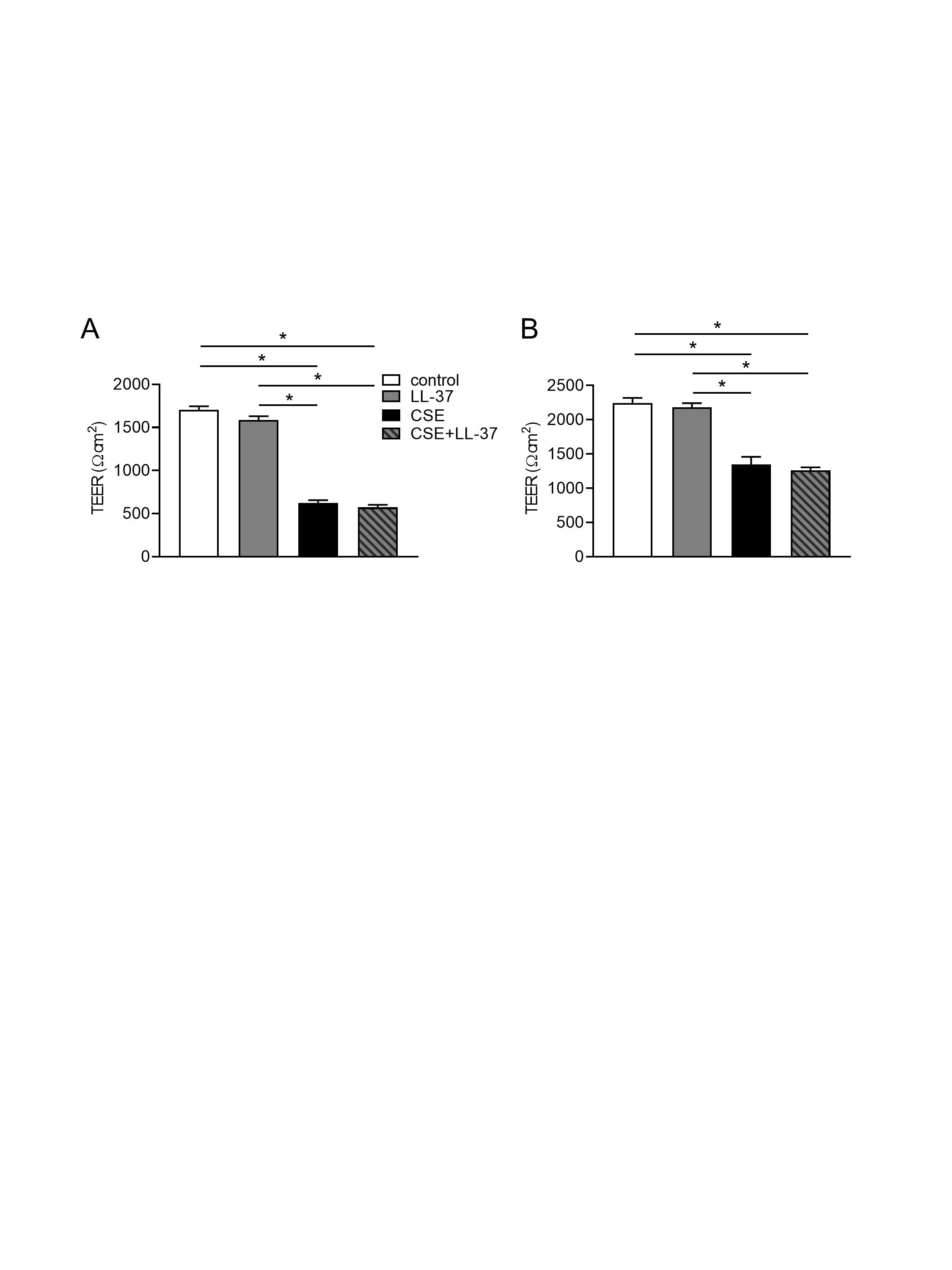

Supplement: Supplementary file 5 — Additional file 5: No protective effects of LL-37 on CSE-induced reduction in TEER disappeared at 48 h after CSE exposure. Calu-3 cells pretreated with or without 20 μg/ml LL-37 were exposed to 10% CSE. A. TEER was measured at 48 h after CSE exposure without retreatment with LL-37. B. Retreatment with 20 μg/ml LL-37 without replacement of the medium was performed at 24 h after CSE exposure and then TEER was measured at 48 h. All results are representative of at least two independent experiments. Data reresent means ± SEM (n = 6–8 per group). *p < 0.001 by one-way ANOVA. [file 12931_2019_1226_MOESM5_ESM.tif]

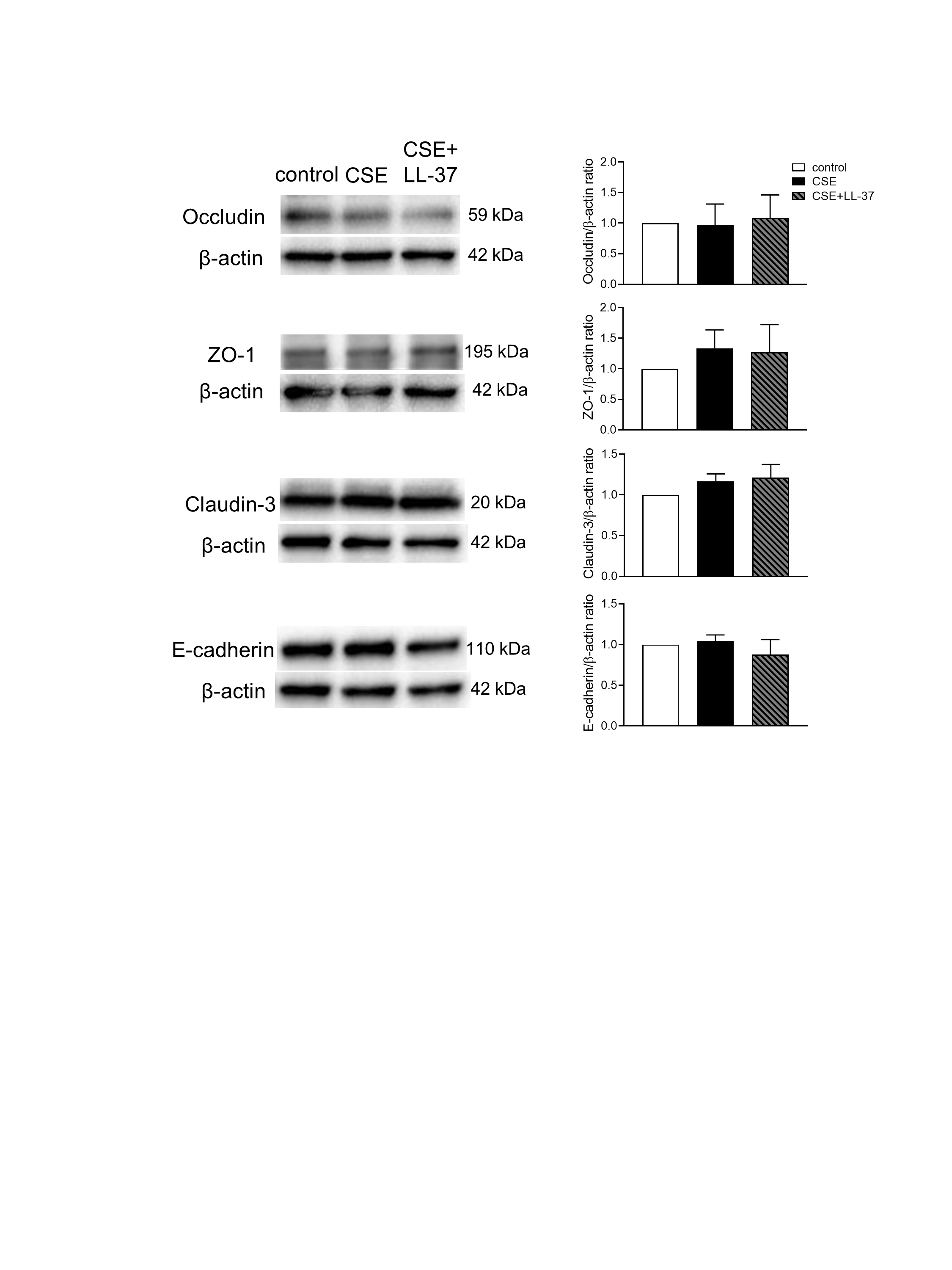

Supplement: Supplementary file 6 — Additional file 6: The effects of LL-37 on protein expression levels for TJ and AJ proteins in Calu-3 cells. Representative western blots showing TJ and AJ proteins in cells pretreated with or without 20 μg/ml LL-37 were exposed to 10% CSE for 24 h. Band intensity was quantitated using densitometry. All results are representative of at least two independent experiments. Data are means ± SEM (n = 3–4 per group). Differences in data were analyzed by one-way ANOVA. [file 12931_2019_1226_MOESM6_ESM.tif]

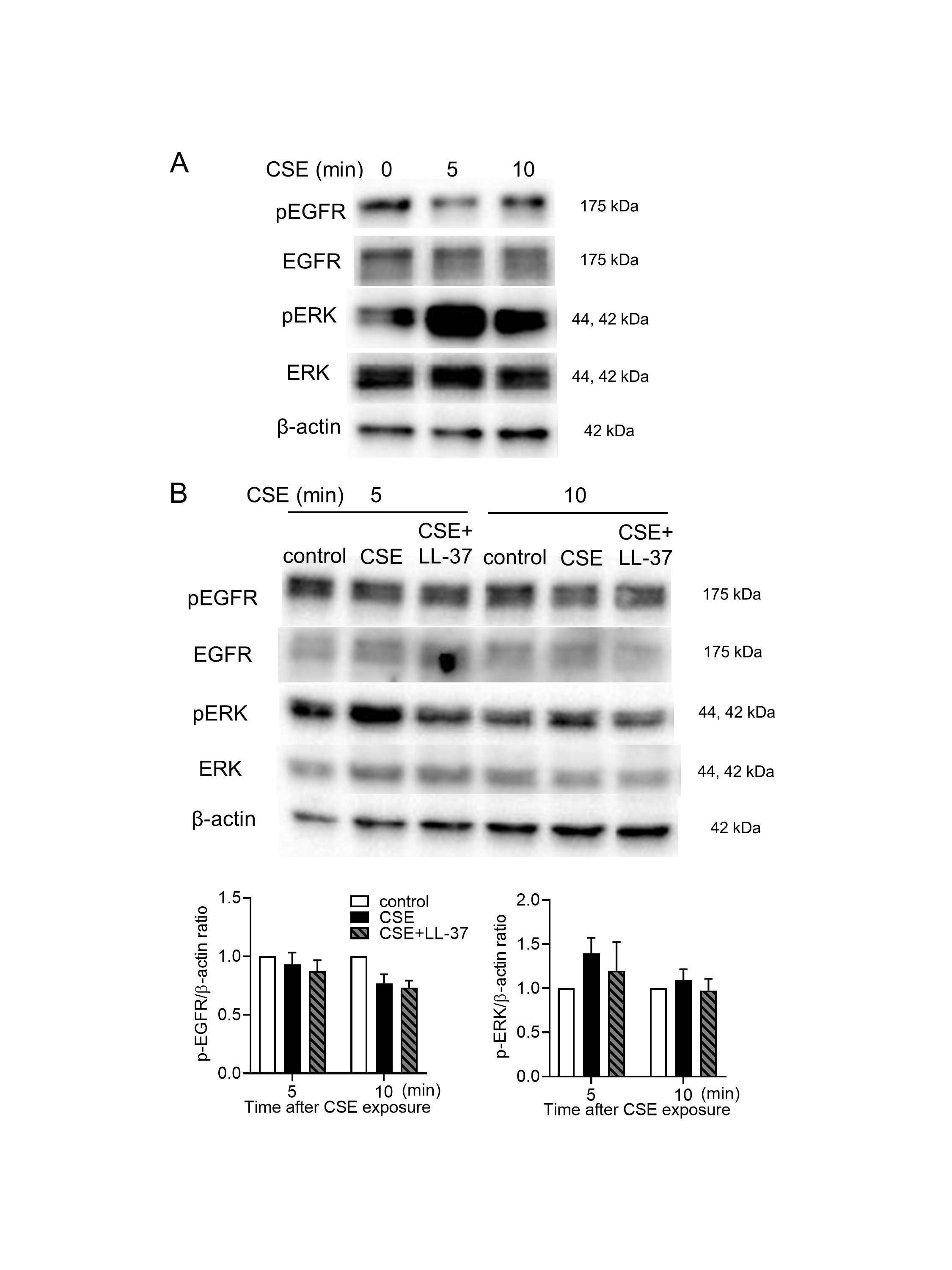

Supplement: Supplementary file 7 — Additional file 7: CSE-induced phosphorylation of ERK and effects of LL-37 on phosphorylation of EGFR and ERK. A. Representative western blots showing phosphorylated EGFR, EGFR, phosphorylated ERK or ERK in Calu-3 cells exposed to 10% CSE for 0, 5 or 10 min after CSE exposure. B. Representative western blots showing phosphorylated EGFR, EGFR, phosphorylated ERK or ERK in Calu-3 cells pretreated with or without 20 μg/ml LL-37 and then exposed to 10% CSE for 5 or 10 min after CSE exposure. Band intensity was quantitated using densitometry. All results are representative of at least two independent experiments. Data represent means ± SEM (n = 4 per group). Differences in data were analyzed by two-way ANOVA. [file 12931_2019_1226_MOESM7_ESM.tif]
