# Supplementary material for: Effects of cigarette smoke on barrier function and tight junction proteins in the bronchial epithelium: protective role of cathelicidin LL-37
Source: Respir Res. 2019 Nov 9;20:251. doi: 10.1186/s12931-019-1226-4 (PMC6842552; doi:10.1186/s12931-019-1226-4)
Supplement: Supplementary file 2 — Additional file 2: Sequences of real-time PCR primers used in this study. [file 12931_2019_1226_MOESM2_ESM.doc]

**Additional file 1 (.txt)**

Real-time PCR primer sequences

*Claudin-1* Sense: 5’ CCGGCGACAACATCGTGAC 3’

Antisense: 5’ CGGGTTGCTTGCAATGTGC 3’

*Claudin-3* Sense: 5’ CGCGAGAAGAAGTACACGG 3’

Antisense: 5’ CCTTAGACGTAGTCCTTGCGG 3’

*Claudin-4*  Sense: 5’ ATCGGCAGCAACATTGTCAC 3’

Antisense: 5’ GCGAGTCGTACACCTTGCAC 3’

*Claudin-7*  Sense: 5’ GGCTTCCTGGCCATGTTTG 3’

Antisense: 5’ GCAAGACCTGCCACGATGAAA 3’

*Claudin-15* Sense: 5’ AGGAAGCAGAGAGACCCACA 3’

Antisense: 5’ AGAACCCCTAGGGAACTGGA 3’

*Occludin*  Sense: 5’ TGCATGTTCGACCAATGC 3’

Antisense: 5’ AAGCCACTTCCTCCATAAGG 3’

*E-cadherin* Sense: 5’ GAAGGTGACAGAGCCTCTGGAT 3’

Antisense: 5’ GATCGGTTACCGTGATCAAAATC 3’

*JAM-A* Sense: 5’ TGCCTATAGCCGAGGCCACT 3’

Antisense: 5’ ATGATAGGCGGTGAGCCGAC 3’

*ZO-1* Sense: 5’ AAGATGTCCGCCAGAGCTGC 3’

Antisense: 5’ AGCGTCACTGTATGTTGTTCCC 3’

*β-actin* Sense: 5’ TGGCACCCAGCACAATGAA 3’

Antisense: 5’ CTAAGTCATAGTCCGCCTAGAAGCA 3’
